# Trends in hospital admissions at a Department for Infectious Diseases in Italy from 1995 to 2011 and implications for health policies

**DOI:** 10.1186/1471-2458-14-980

**Published:** 2014-09-20

**Authors:** Giovanna Orlando, Guido Gubertini, Cristina Negri, Massimo Coen, Elena Ricci, Massimo Galli, Giuliano Rizzardini

**Affiliations:** Department of Infectious Disease I, L Sacco University Hospital, Milan, Italy; Department of Infectious Disease III, L Sacco University Hospital, Milan, Italy; STD Unit, L Sacco University Hospital, Via GB Grassi, 74, 20157 Milan, Italy

**Keywords:** Epidemiology, Infectious diseases, Hospitalization, ICD-9, Major diagnostic categories, Public health policy, Surveillance

## Abstract

**Background:**

Interactions among several environmental, behavioral, social, and biological variables contribute to the epidemiology of infectious diseases (IDs) and have an impact on the healthcare system and hospitalizations. We evaluated trends in ID hospitalizations at our Department for Infectious Diseases in the last two decades to aid decision-makers in defining appropriate healthcare strategies.

**Methods:**

The discharge diagnoses of all patients admitted to the ID Department of L Sacco University Hospital between 1995 and 2011 were classified by the International Classification of Diseases (ICD-9) grouped in Major Diagnostic Categories (MDC). Linear regression was used to determine the trends in hospitalizations for each MDC. Estimates of the average annual change were based on the slope of the regression line.

**Results:**

A sharp decline in HIV/AIDS cases (−22.5 +/−6.0 cases per calendar year), and an increase in admissions for respiratory, cardiovascular, renal and musculoskeletal infections were recorded. The mean age of the patients increased by 1.2 years (+/−0.049) for each calendar year of observation (linear trend, p < 0.0001), increasing from 37.02 +/−11.91 years in 1995 to 56.02 +/−19.62 years in 2011 (p < 0.0001). The mean number of comorbidities per patient increased significantly over time (Mann–Whitney *U* test, p = 0.0153). From 1998/1999 to 2010/2011 the hospital length of stay (LOS) increased for cardiovascular, digestive system, musculoskeletal, and skin/subcutaneous infections, and infectious and parasitic diseases (p < 0.01). The rate of hospital stay over threshold (HSOT) increased in the last 5 years by 1.12% for every 10-year age group.

**Conclusions:**

Older age, a higher number of comorbidities, a longer hospital LOS for certain conditions, and a higher rate of HSOT characterize the patients admitted to this ID department in recent years. Despite progress in treatment and management, infectious diseases continue to be a major threat to human health. The current challenge for ID departments is the treatment of complex cases, often associated with chronic diseases in elderly patients. Continuous monitoring at a local and national level will allow early identification of changes in the epidemiological patterns of IDs and provide information for healthcare system planning.

## Background

The epidemiology of infectious diseases (IDs) is a composite of several environmental, behavioral, social, and biological variables, including the selective effect of drugs or vaccines, and other unpredictable events that can change over time and have potentially important effects on global health. It is estimated that IDs are the second leading cause of death worldwide after cardiovascular diseases [1]. Respiratory infections, diarrheal diseases, human immunodeficiency virus acquired immunodeficiency syndrome (HIV/AIDS), tuberculosis (TB), and malaria account for 18.3% of all causes of death in 2004, although major differences in their ranking exist between high- and low-income countries [1].

Communicable diseases remain a major health threat in Europe, although there are wide differences among the World Health Organization European regions. In the “Health at a Glance: Europe 2012” more than 6,000 new hepatitis B cases, with a mean rate of 2.0/100,000 population, and 27,000 newly diagnosed HIV infections were reported by EU member states [2].

The public health importance of IDs and communicable diseases is mostly related to the continuing threat of epidemic/pandemic events), the emergence of new diseases and re-emergence of old diseases, the threat of imported diseases or pathogens, and the emergence of multidrug or pan-drug resistant organisms [3–13].

Hospital ID departments are a useful source of surveillance data that can indicate changing healthcare requirements. For example, a great effort was made In Italy in the late 1980s to tackle the AIDS epidemic, with an increase in the overall number of hospital beds to deliver services to HIV-infected inpatients. Twenty years on, the healthcare needs of HIV-infected patients have changed, and they are now mainly followed up as outpatients. In 2010, the overall rate of hospital discharge of HIV/AIDS patients was 3.9/10,000 compared with 29.87/10,000 inhabitants for other IDs excluding AIDS (ICD-9 codes 001–139) [14]. Over the same period, however, there has been an increasing need for hospital admission for several other infectious or communicable diseases.

The rapid and often unpredictable changes in the epidemiology of IDs, the significant burden on public health and the global economy, the breakthroughs in the field of prevention, and the new treatment opportunities for acute and chronic infectious diseases require ongoing evaluation in order to formulate appropriate healthcare and public health strategies. In particular, several structural and organizational choices for ID departments and related services must be made in relation to changing healthcare needs and priorities.

This study determined the temporal trends of hospitalizations in an ID department in the period 1995–2011 after the introduction of hospital funding on a “per case” basis (1995), classified according to the Diagnosis Related Group (DRG) system in Italy. The aim of the study was to evaluate changes in healthcare needs of patients admitted to an ID department and to provide some evidence for structural and organization changes in healthcare delivery for patients suffering from infectious or communicable diseases.

## Methods

We retrospectively analyzed the discharge diagnosis of all patients admitted to the ID Department of L Sacco University Hospital between January 1995 and December 2011. This Department is a referral center for acute and highly communicable infectious diseases in Northern Italy. It provides the clinical care and follow-up of patients older than 18 years suffering from viral, bacterial, and parasitic diseases. Patients affected by gynecological infections are admitted to Gynecology/Obstetrics Departments. The ID department includes outpatient wards and day hospital services, a specific emergency room, and wards for hospital admissions (87 beds). The ID specialists on 24-hour duty at the Emergency Department of the Hospital decide on the admission of patients with suspected infectious or communicable diseases to their own Department according to triage rules, and to epidemiological and/or clinical priorities. Patients who need intensive care are primarily admitted to the intensive care unit and not to the ID department.

From the patient records, we collected demographic information, up to six discharge diagnoses and up to six procedures per hospitalization, the DRG, and the number of days of in-hospital care. The ICD-9 codes were classified into 25 groups (Major Diagnostic Categories (MDC) version 24) of related conditions which accounted for all the DRGs at discharge, and the MDCs were evaluated for their trends over time.

As surrogate markers of clinical complexity we used the number of ICD-9 codes for diseases (ICD-9c) and procedures (ICD-9p) notified in the hospital discharge card, the hospital length of stay (LOS), and the hospital stay over threshold (HSOT; days of hospitalization over the limit for the specified condition defined by national average data).

We examined temporal trends of hospitalization according to the demographic characteristics of the population: sex, age, and age group.

### Statistical analysis

The D’Agostino and Pearson omnibus normality test was used for the descriptive variables to assess their distribution. The unpaired *t*-test with Welch correction and the Mann–Whitney *U* test were used to compare the mean or medians of parametric and non-parametric values respectively. A p-value < 0.05 was considered statistically significant.

Linear regression was used to estimate the trends in hospitalization for each MDC according to sex, age, geographical origin of the patients, number of ICD-9c and ICD-9p, and in-hospital LOS. Estimates of the mean annual change were based on the slope of the regression line. Analyses were conducted with the GraphPad Prism version 4.02 for Windows (GraphPad Software, San Diego, CA, USA).

Multivariate linear regression analysis was performed for hospital LOS, including age group and sex in the model. The association between HSOT and demographic and clinical characteristics was evaluated by logistic regression. To account for the effects of several potential confounding factors, we used unconditional multiple logistic regression, with maximum likelihood fitting, to obtain odds ratios and their corresponding 95% confidence intervals. Included in the regression equations were sex, geographical origin of the patients, age group, and number of ICD-9c and ICD-9p. Multivariate analyses were performed using the SAS/STAT software package version 9.1 (Cary, NC, USA).

The study was approved by the local Ethical Committee on July 2013 (Protocol N 509/2013/73/ap) and was conducted in accordance with the Helsinki declaration.

## Results

Between January 1995 and December 2011, there were 26,253 admissions to the ID department. The mean number of annual hospital admissions was 1544 ± 200.4. In total, there were 17,719 (67.49%) males and 8,534 females; the median age was 42.4 years (interquartile range (IQR): 34.3–57.7 years); median hospital LOS was 10 days (IQR: 6–19 days). Foreign-born patients accounted for 19.67% of the total admissions.

### Trend in hospital admissions by MDC

Table 1 presents the crude number of admissions for specified conditions in the whole period, in the first and last year of observation, the slope of linear regression for trend, and its deviation from zero. Overall, the number of admissions for MDC-25 (HIV/AIDS) was by far the highest (44.4%), followed by MDC-07 (diseases and disorders of liver, gallbladder and pancreas) (12.68%), MDC-18 (infectious and parasitic diseases) (11.34%), and MDC-04 (diseases and disorders of the respiratory system) (9.96%). Within MDC-07, acute and chronic viral liver diseases (DRG 205, 202, 206) accounted for 87.14% of the cases; within MDC-18, infectious diseases, sepsis, viral infections and fever of unknown origin (DRG 423, 416, 421, 420, 576) accounted for 89.89% of cases; within MDC-04, pneumonia, other respiratory infections, and chronic obstructive pulmonary diseases (DRG 89, 90, 79, 80, 88) accounted for 82.52% of the cases.

**Table 1:** **Overall number of admissions for specified conditions (MDC), and number of admissions in the first and last year of observation**

		Admissions						Unadjusted model		Adjusted model	
MDC	Description	Period 1995-2011		1995		2011		Slope ± SD	Deviation from zero - p value	Slope ± SD	Deviation from zero - p value sex- and age-adjusted
		N	%	N	%	N	%				
01	Diseases and disorders of the nervous system	709	2.70	17	1.58	28	1.76	−0.61 ± 0.96	0.53	−0.61 ± 0.66	0.37 ^a^
02	Diseases and disorders of the eye	62	0.24	3	0.28	8	0.50	0.093 ± 0.16	0.57	/	/
03	Diseases and disorders of the ear, nose, mouth and throat	339	1.29	8	0.74	24	1.50	**1.0 ± 0.39**	**0.02**	**0.81 ± 0.29**	**0.006** ^**b**^
04	Diseases and disorders of the respiratory system	2614	9.96	8	0.74	235	14.73	**15 ± 1.9**	**<0.0001**	**14.5 ± 1.48**	**<0.0001** ^**a,b**^
05	Diseases and disorders of the cardiovascular system	443	1.69	2	0.19	41	2.57	**1.7 ± 0.61**	**0.014**	**1.51 ± 0.45**	**0.001** ^**a,b**^
06	Diseases and disorders of the digestive system	953	3.63	9	0.83	76	4.76	2.6 ± 1.3	0.064	**1.87 ± 0.84**	**0.03** ^**b**^
07	Diseases and disorders of liver, gallbladder and pancreas	3329	12.68	55	5.10	159	9.97	0.92 ± 3.0	0.076	0.92 ± 2.32	0.69 ^a^
08	Diseases and disorders of the musculoskeletal system and connective tissue	629	2.40	5	0.46	60	3.76	**4.3 ± 0.56**	**<0.0001**	**3.85 ± 0.59**	**<0.0001** ^b^
09	Diseases and disorders of the skin, subcutaneous tissue and breast	896	3.41	9	0.83	62	3.89	1.5 ± 1.2	0.23	1.03 ± 0.80	0.20 ^b^
10	Endocrine, nutritional and metabolic diseases and disorders	72	0.27	1	0.09	6	0.38	0.076 ± 0.17	0.65	/	/
11	Diseases and disorders of the kidney and urinary tract	432	1.65	1	0.09	52	3.26	**2.3 ± 0.45**	**0.0001**	**1.91 ± 0.49**	**0.0003** ^b^
12	Diseases and disorders of the male reproductive tract	65	0.25	1	0.09	9	0.56	0.22 ± 0.13	0.098	/	/
13	Diseases and disorders of the female reproductive tract	31	0.12	1	0.09	1	0.06	−0.014 ± 0.093	0.88	/	/
14	Pregnancy, childbirth and puerperium	2	0.01	0	/	0	/	/	/	/	/
15	Diseases and disorders of the neonatal period	1	0.00	0	/	0	/	/	/	/	/
16	Diseases and disorders of blood, myelopoietic and immune system	238	0.91	10	0.93	17	1.07	0.53 ± 0.30	0.10	0.53 ± 0.30	0.18
17	Diseases and myeloproliferative disorders and poorly differentiated tumors	380	1.45	1	0.09	15	0.94	0.50 ± 0.66	0.46	−0.32 ± 0.44	0.46 ^**a,b**^
18	Infectious and parasitic diseases (systemic or unspecified sites)	2978	11.35	23	2.13	333	20.88	**14 ± 2.0**	**<0.0001**	**14.00 ± 1.68**	**<0.0001** ^**a**^
19	Diseases and Mental Disorders	28	0.11	1	0.09	1	0.06	−0.21 ± 0.17	0.25	/	/
20	Abuse of alcohol / drug induced organic mental disorders	27	0.10	1	0.09	1	0.06	−0.33 ± 0.20	0.14	/	/
21	Injury, poisoning and toxic effects of drugs	127	0.48	2	0.19	9	0.56	−0.099 ± 0.32	0.76	−0.08 ± 0.19	0.63
22	Burns	2	0.01	0	/	0	/	/	/	/	/
23	Factors influencing health status and use of health services	79	0.30	21	1.95	1	0.06	**−0.79 ± 0.28**	**0.014**	**/**	**/**
24	Multiple relevant trauma	3	0.01	0	/	0	/	/	/	/	/
25	HIV infection	11657	44.41	897	83.21	452	28.34	**−23 ± 6.0**	**0.002**	**−22.52 ± 9.56**	**0.02** ^**a,b**^
	other DRG	81	0.31	1	0.09	2	0.13	0.16 ± 0.15	0.28	−0.19 ± 0.55	0.73 ^**a**^
	Pre MDC	71	0.27	1	0.09	3	0.19	−0.24 ± 0.21	0.28	/	/

Mean annual change based on the slope of the regression line in the crude and multivariate adjusted models.

MDC with less than 100 observations have been excluded in the multivariate model adjusted for sex and class of age.

^a^= significantly associated to sex; ^b^= significantly associated to age (≥45 years). Bold indicates statistically significant results.

*MDC* Major Diagnostic Category, *DRG* Diagnosis-Related Group, *SD* standard deviation.

In the model adjusted for sex and age (>45 years) a significant increase in admissions for infectious diseases of the ear, nose, mouth and throat (MDC-03), respiratory infections (MDC-04), infections of the cardiovascular system (MDC-05), infections of musculoskeletal/connective tissue (MDC-08), infections of the kidneys and urinary tract system (MDC-11), and unspecified infectious or parasitic diseases (MDC-18) was found over the period, while there was an overall decrease in HIV/AIDS admissions (−22.52 ± 6.0 cases per calendar year). All significant variations in hospital admissions were associated with age > 45 years except for MDC-18 (infectious and parasitic diseases), which had variations associated with female sex.

**Figure 1:**
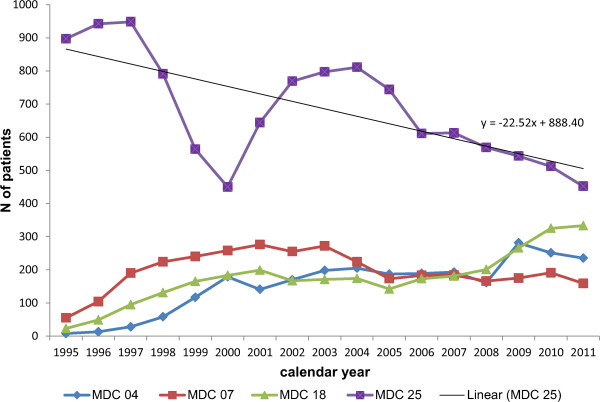
**Temporal trend in hospital discharge for selected MDC.**

The graph of hospital admissions for HIV/AIDS showed a rapid decline after 1996 (Figure 1), coinciding with the widespread introduction of highly active antiretroviral therapy (HAART). Nowadays, although HIV/AIDS remains the main cause of admission to the ID department, the proportion of admissions has fallen from 83.13% to 28.34% of all causes of admission, while the proportion of admissions for other MDC has progressively increased.

### Demographic data

In 1995, when HIV/AIDS infection was the major cause of hospital admission to our ID department, the male/female ratio of admitted patients was 2.9:1. Over time a progressive narrowing of the gap was observed, for an increase in admissions of female patients (admissions slope ± standard deviation (SD), +19.05 ± 3.54; deviation from zero, p < 0.0001) while the admissions for males remained constant.

The number of foreign-born patients was stable during the period (slope, +7.82 ± 3.83; no significant deviation from zero) while the number of Italian-born patients increased by +13.28 ± 5.41 for each calendar year (deviation from zero, p = 0.027).

The mean age of the patients increased by +1.2 ± 0.049 years per calendar year (linear trend, p < 0.0001) from 37.02 ± 11.91 years in 1995 to 56.02 ± 19.62 years in 2011 (unpaired *t*-test with Welch’s correction, p < 0.0001).

**Figure 2:**
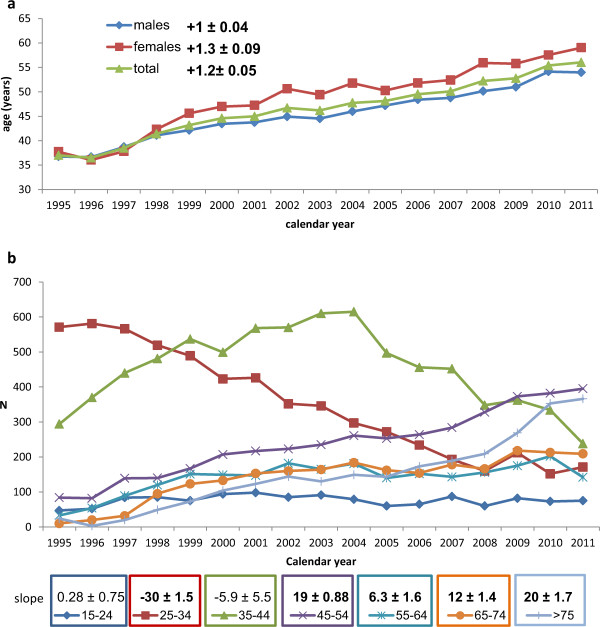
**Mean age of patients admitted to the Infectious Diseases Department between 1995 and 2011, with slope of the regression line according to sex (a), and trends by age group and related slopes of the regression lines (b).**

In the analysis by age group, the overall increase in the mean age was a result of two opposite trends: an increase in hospital admissions of the oldest patients (slope, +20 ± 1.7 patients/year for those aged >75 years and +12 ± 1.4 patients/year for those aged 65–74 years), and a decrease in admission of patients aged 25–34 years (slope, −30 ± 1.5 patients/year). In the last year of observation, the 45–54-year and the >75-year age groups were the ages most frequently admitted (Figure 2).

### Trend in severity data and in hospital LOS

The mean number of ICD-9c/patient decreased from 3.1 ± 1.06 in 1995 to 2.7 ± 1.13 in 1999, then it increased again to reach 3.1 ± 1.55 in 2011, with an overall significant increasing trend (slope, + 0.015 ± 0.01; deviation from zero, p = 0.029). The number of ICD-9p/patients increased from 0.3 ± 0.62 in 1995 to 0.9 ± 1.3 in 2011 (slope, +0.035 ± 0.01; deviation from zero, p < 0.0001).

**Figure 3:**
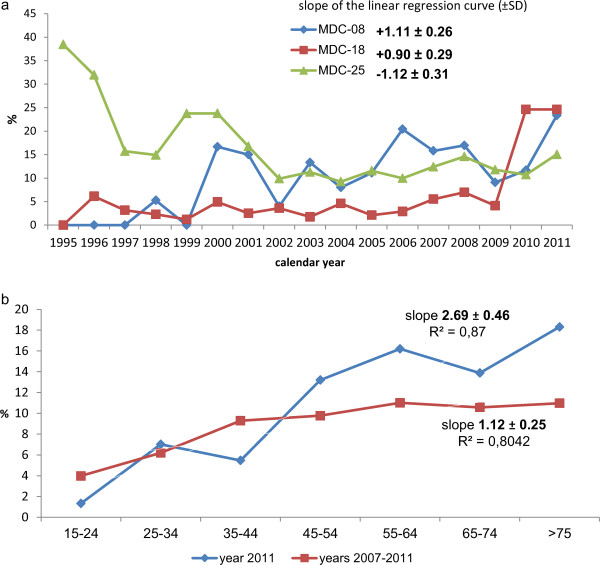
**Rate of hospital stay over threshold (HSOT) for specified conditions by year (1995–2011) (a), and by age group in 2011 and in the last 5 years of observation (2007–2011) (b).**

The mean hospital LOS for all causes declined significantly from 25.17 ± 22.65 days to 15.33 ± 15.99 days (mean reduction, −0.43 ± 0.17 days/year). A dramatic drop in LOS was registered between 1996 and 1997, when major changes in the Italian healthcare system were implemented. To exclude this administrative effect, the analysis of trend of hospital LOS was restricted to the period 1998–2011. A significant decrease in hospital LOS for MDC-07 and −25 was observed. In the same period, hospital LOS increased significantly for MDC-05, −06, −08, −09, and −18 (Mann Whitney *U* test, p < 0.01). The slope of the linear regression was significantly different from zero in the model, adjusted for sex and age group, for infections of the cardiovascular system (MDC-05), liver (MDC-07), infectious and parasitic diseases (MDC-18), and HIV/AIDS (MDC-25) (Table 2). A high number of HSOT was observed for some common conditions: 81 cases (12.88%) for diseases of the musculoskeletal system and connective tissue (MDC-08), 247 cases (8.29%) for infectious and parasitic diseases (MDC-18) and 1996 cases (17.12%) for HIV/AIDS (MDC-25). For these conditions, the trend of HSOT tended to decrease for HIV/AIDS and to increase for MDC-18 and for MDC-08 (Figure 3a). The rate of HSOT was significantly related to age: in the last 5 years (2007–2011), the rate of HSOT increased by 1.12% for each 10-year age group and in the last year of observation this increase was particularly high (2.69% for each 10-year age group) (Figure 3b).

**Figure 4:**
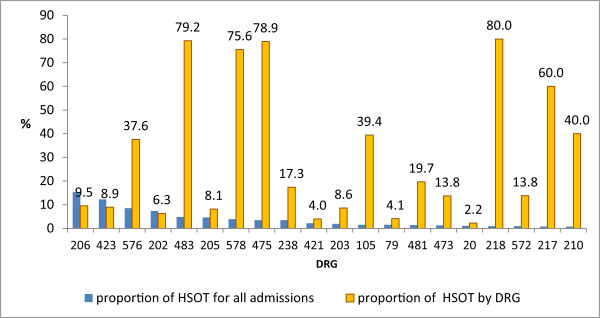
**Proportion of HSOT by the first 20 DRG with the highest proportion of prolonged hospitalization for all patients excluding HIV/AIDS.**

After excluding HIV-infected patients, the proportion of patients with HSOT was 5.96%. The commonest primary diagnosis of patients with HSOT was liver cirrhosis, followed by other infectious and parasitic diseases and sepsis. Figure 4 illustrates the proportion of HSOT in patients with the 20 most common DRGs.

**Table 2:** **Comparison of median and IQR hospital LOS for specified conditions (MDC) for the years 1998/1999 and for the years 2010/2011; mean annual change in the period 1998–2011 based on the slope of the regression line adjusted for sex and age group, and its deviation from zero**

MDC	Description	N of patients (1998–2011)	Median hospital LOS (IQR) in 1998/1999	Median hospital LOS (IQR) in 2010/2011	Mann Whitney U test	Slope (±S D)	Deviation from zero p-value, sex- and age-adjusted
	All the patients	22649	10 (6–18)	10 (6–18)	0.20	−0.02 ± 0.02	0.36b
01	D&D of the nervous system	645	12 (7–18)	14 (8–20.5)	0.23	0.23 ± 0.12	0.06b
02	D&D of the eye	59	8 (5.5-18.5)	9 (5–18)	1.00	/	/
03	D&D of the ear. nose. mouth and throat	327	6 (4.5-8)	6 (3–10)	0.86	0.003 ± 0.09	0.97b
04	D&D of the respiratory system	2565	10 (6–17)	9 (6–15)	0.42	−0.07 ± 0.06	0.24b
05	D&D of the cardiovascular system	428	9.5 (5–19)	16.5 (9–42)	**0.0006**	1.47 ± 0.24	**<0.0001** ^**a**^
06	D&D of the digestive system	923	8 (4–13)	11 (6–17)	**0.0015**	0.002 ± 0.09	0.98b
07	D&D of liver. gallbladder and pancreas	2980	11 (6–18)	9 (5–14)	**0.0018**	−0.24 ± 0.05	**<0.0001** ^**b**^
08	D&D of the musculoskeletal system and connective tissue	619	15 (8–22)	19.5 (11.5-33)	**0.0091**	0.2 ± 0.21	0.32b
09	D&D of the skin. subcutaneous tissue and breast	865	7 (4–11)	10 (6–17)	**0.0001**	0.17 ± 0.09	0.06b
10	Endocrine. nutritional and metabolic diseases and disorders	70	8 (6–11.5)	8.5 (4.5-10.5)	0.95	/	/
11	D&D of the kidney and urinary tract	423	8 (5.5-14.5)	9 (5–15)	0.95	−0.20 ± 0.15	0.17b
12	D&D of the male reproductive tract	62	8 (5.5-14)	8 (5.5-10.5)	0.90	−0.47 ± 0.39	0.24
13	D&D of the female reproductive tract	29	12 (3.5-21.5)	14 (8.5-20.5)	0.79	/	/
14	Pregnancy, childbirth and puerperium	2	/	/	/	/	/
15	D&D of the neonatal period	1	/	/	/	/	/
16	D&D of blood. myelopoietic and immune system	214	10 (7-229	11 (6.5-18)	0.61	−0.25 ± 0.18	0.16
17	Diseases and myeloproliferative disorders and poorly differentiated tumors	374	4 (2–15)	9.5 (5–16.5)	0.06	0.003 ± 0.20	0.99
18	Infectious and parasitic diseases (systemic or unspecified sites)	2811	7 (4–11)	9 (5–20)	**<0.0001**	0.26 ± 0.05	**<0.0001** ^**b**^
19	Diseases and Mental Disorders	27	7 (4–14)	/	/	/	/
20	Abuse of alcohol / drug induced organic mental disorders	27	3.5 (2.5-8.5)	/	/	/	/
21	Injury. poisoning and toxic effects of drugs	124	5.5 (3–10)	8 (4.5-28)	0.06	0.75 ± 0.35	**0.034**
22	Burns	2	/	/	/	/	/
23	Factors influencing health status and use of health services	53	7 (3–9)	/	/	/	/
24	Multiple relevant trauma	3	/	/	/	/	/
25	HIV infection	8870	12 (7–22)	10 (6–18)	**0.0044**	−0.19 ± 0.04	**<0.0001**
	Pre MDC	76	53 (11–69)	55.5 (24.5-74)	0.95	/	/
	other DRG	70	18 (17–22)	/	/	/	/

MDC with less than 100 observations have been excluded in the multivariate model adjusted for sex and age group.

^a^= significantly associated with sex; ^b^= significantly associated with age (≥45 years).

*LOS* length of stay, *D&D* diseases and disorders, *IQR* interquartile range.

Bold indicates statistically significant results.

*MDC* Major Diagnostic Category, *SD* standard deviation.

Patients with HSOT (excluding HIV/AIDS) were significantly older than patients who had a shorter hospital stay (median age: 59.12 years (IQR 39.8–74.23) vs 50.33 years (IQR 34.31–69.92). These patients also had a significantly higher number of ICD-9c and ICD-9p. In the univariate model, the odds ratio of HSOT was significantly higher for foreign-born patients, for patients older than 45 years, and for those with more than one ICD-9c or at least one ICD-9p on the discharge card. In the multiple logistic regression analysis, the number of ICD-9c and ICD-9p remained significantly associated with a longer hospital stay, while the geographical origin of the patient and the age group showed no significant association (Table 3).

**Table 3:** **Univariate and unconditional multiple logistic regression analysis of the association of demographic and clinical characteristics of patients admitted for all MDCs (excluding HIV/AIDS) with long hospital stay**

	Hospital stay over threshold (HSOT)	Hospital stay in threshold	p value	Crude OR (95%CI)		Adjusted ^b^OR (95%CI)	
Males	493	7861	<0.41^a^	1			
Females	315	5283		1.01	0.87-1.17	0.88	0.74-1.03
Italian born people (Ref)	660	12181	**<0.0001** ^**a**^	1		1	
Foreign born people	148	956		**2.86**	**2.36-3.45**	1.22	0.96-1.54
Class of age			**<0.0001** ^**a**^				
15-24 years (ref)	48	1079		1		1	
25-34 years	116	2452		1.13	0.76-1.69	1.15	0.74-1.78
35-44 years	112	2485		1.19	0.80-1.77	0.85	0.54-1.32
45-54 years	123	1689		**2.02**	**1.36-2.99**	1.19	0.76-1.84
55-64 years	119	1656		**1.92**	**1.30-2.85**	1.02	0.65-1.59
65-74 years	167	1980		**2.35**	**1.61-3.42**	1.10	0.72-1.70
> 74 years	184	2279		**2.34**	**1.61-3.40**	1.08	0.70-1.75
N of ICD-9 codes							
1 (ref)	118	4063	**<0.0001** ^**a**^	1		1	
2	122	3755		**1.50**	**1.11-2.02**	1.16	0.85-1.58
3	163	2684		**2.83**	**2.14-3.75**	**1.87**	**1.38-2.54**
4	169	1709		**4.74**	**3.58-6.28**	**2.99**	**2.20-4.07**
5	102	713		**7.04**	**5.17-9.59**	**4.16**	**2.95-5.87**
6	191	750		**12.54**	**9.49-16.56**	**8.02**	**5.89-10.93**
N of ICD-9 procedures							
0 (ref)	313	8627	**<0.0001** ^**a**^	1		1	
1	164	2500		**1.96**	**1.59-2.41**	**1.89**	**1.52-2.35**
2	106	1321		**2.37**	**1.86-3.02**	**1.87**	**1.43-2.44**
3	110	636		**5.41**	**4.25-6.87**	**4.10**	**3.16-5.34**
4	57	321		**5.43**	**4.06-7.56**	**4.08**	**2.92-5.68**
5	54	169		**10.06**	**7.23-14.0**	**6.59**	**4.55-9.55**
6	65	148		**13.82**	**10.07-18.98**	**7.97**	**5.61-11.33**

^a^χ^2^ test; ^b^all variables analyzed in the univariate analysis were included in the regression equation.

*OR* odds ratio, *CI* confidence interval. Bold indicates statistically significant results.

## Discussion

In the whole period considered a significantly higher rate of hospital admissions in the ID Department for MDC-25 (HIV/AIDS), MDC-07 (Diseases and Disorders of liver, gallbladder and pancreas), MDC-18 (Infectious and parasitic diseases), and MDC-04 (Diseases and disorders of the respiratory system) can be observed if compared with the rate of admissions registered in Italy in 2010 in all public hospitals so as they can represent specific conditions for the ID Departments [14].

This study showed a marked change in the type of ID which resulted in hospital admission during the period 1995–2011. The most striking change was the decrease in hospital admissions for HIV/AIDS from 84.4% of all hospital ID admissions in 1995 to 28.3% in 2011. This was primarily due to the changes in the clinical characteristics of HIV/AIDS after the introduction of HAART in 1996. The disease was initially characterized by acute and life-threatening opportunistic infections and cancers needing hospitalization in acute care hospitals; after the widespread use of HAART in the industrialized world, HIV/AIDS changed to a chronic disease mainly managed in outpatient settings. The HIV Research Network described a decrease in the all-cause hospitalization rate for persons living with HIV/AIDS from 35/100 person-years to 27/100 person-years from 2002 to 2007, and an increase in hospital admission rates for cardiovascular, renal and pulmonary conditions associated with HIV infection [15–18]. However, the drop in admissions for HIV infection was quickly replaced with an increase in admissions for other infectious diseases.

The second most important change observed in this study was the aging of the admitted patients. In the 1990s, the great majority of patients were young HIV-infected patients, but now patients are more likely to be middle-aged or old patients with chronic-degenerative comorbid conditions. The most marked increase in hospital admission was recorded for patients aged > 75 years, with a mean increase in ID admissions of more than 20 cases per year.

Aged patients and comorbidities could, in part, explain the increase over time of the complexity of admitted patients, with increases in the mean number of ICD-9 codes and procedures, hospital LOS, and rate of HSOT. One of the limits in the definition of the overall complexity of the patients admitted is the lack, in the hospital discharge card, of a reliable method for the evaluation of this parameter. It would be of interest to include the age-adjusted Charlson Comorbidity Index in the hospital discharge card, not only for clinical and epidemiological reasons, but also to adjust for the overall clinical complexity of cases the financial reimbursement system (DRG system).

The decrease in LOS over the whole period is apparently in contrast with the higher clinical complexity measured by the number of ICD-9c and ICD-9p for each patient. The greatest difference was observed in the years 1996–1997 when the mean LOS fell from 27.13 days to 16.33 days and thereafter remained stable. The dramatic drop was related to the changes in healthcare funding for hospitalizations rather than to changes in clinical/epidemiological parameters. The Italian Finance Act No 724/94 introduced hospital funding on a “per case” basis from calendar year 1995. The mean adjustment period of the system to reach equilibrium was 3 years. After this period a significant decrease in the total number of in-hospital beds for acute diseases (−24.8% from 1995 to 2003), an increase in the activities volume, and a decrease in LOS was observed in all Italian public hospitals [19]. After 1997, we found that the overall LOS remained stable, but there were significant differences among MDCs: the hospital LOS increased significantly for MDC-05, −06, −08, −09, and −18, and decreased for MDC-07 and −25.

In the adjusted model for sex and age group, an increasing trend for infections of the cardiovascular system, and for infections and parasitic diseases, and a decreasing trend for infectious diseases of the liver, and for HIV/AIDS was confirmed.

A strong association was found between older age and delayed discharge, with longer LOS and higher rates of HSOT. An association between older age and comorbid conditions has already been reported, with longer hospitalization in patients with community-acquired pneumonia, chronic obstructive pulmonary diseases, and post-surgical complications [20–22]. In our analysis, long hospitalization was related to a greater disease burden measured by the number of ICD-9c and ICD-9p, which mainly occurred in older patients. Our data confirm a high rate of HSOT in patients with complicated or post-surgical infections, or in severely immunocompromised patients with non-AIDS-related conditions (bone marrow transplant, leukemia). In a previous retrospective analysis, prolonged hospitalizations were observed in elderly patients with a primary diagnosis of sepsis (43.1%) and neurological disorders (16.1%); the two most common factors contributing to a delay in discharge were social issues (39.4%) and sepsis (34.3%), which was mostly hospital-acquired (78,7%) [23]. Longer hospitalization not only increases costs, but it is also associated with increased risk of hospital-acquired infections.

One of the limitations of our retrospective analysis of hospital discharge records was the inability to define the proportion of nosocomial infections, or infections from highly resistant pathogens which may have contributed to an extended hospital stay. The analysis could not evaluate the extent to which social issues contributed to delayed discharge, and which may be particularly relevant in foreign-born patients and the elderly. In Italy, the proportion of the population older than 75 years is about 10%, one quarter live alone, and they account for 17.7% of healthcare expenditure [24]. With progressive aging of the population and longer life expectancy, the demand for healthcare in the elderly will increasingly impact on the healthcare system, and the need for post-acute treatment will become increasingly important.

In recent years, there has been an ongoing attempt to move toward healthcare outwith hospitals. The sub-acute care units, recently introduced in Italy as part of the reorganization of the healthcare system, meet the need for the post-acute management of certain conditions requiring long-term anti-infective treatments (osteomyelitis, prosthesis infections, wound and ulcer infections, endocarditis, etc.). However a more comprehensive reorganization of the system outside acute care hospitals is probably needed for the control of acute and chronic ID including constant surveillance of the microbial landscape, public health efforts to contain emerging or re-emerging threats, efficient analysis of surveillance data, and new discoveries in ID therapeutics.

## Conclusions

The social and demographic changes in the population, and the changes in the epidemiological patterns of IDs, outlined in this study, need constant and careful monitoring at local and national levels. Despite progress in their treatment and management, IDs continue to be a major threat to human health even in industrialized countries. We believe that appropriately equipped medical centers with high expertise will still be essential in the future. In addition team-based primary care models, including ID specialists to monitor anti-infective drugs and the containment of drug resistance, post-acute care services, improvements in non-institutional care for seniors with chronic infective and non-infective conditions, and, eventually, expanded use of electronic e-health services are essential to properly control IDs and healthcare costs.

## Abbreviations

ID: Infectious diseases
ICD-9: International classification of the diseases
MDC: Major diagnostic categories
LOS: Length of stay
HSOT: Hospital stay over threshold
HIV/AIDS: Human immunodeficiency virus//acquired immunodeficiency syndrome
TB: Tuberculosis
DRG: Diagnosis related groups
BLS4: Bio safety level 4
MDC: Major diagnostic categories
ICD-9p: International classification of the diseases procedures
ICD-9c: International classification of the diseases conditions
95%CI: 95% confidence interval
IQR: Interquartile range
OR: Odds ratio
NS: Not significant
HAART: Highly active antiretroviral treatment.
